# Supplementary material for: triact package for R: analyzing the lying behavior of cows from accelerometer data
Source: PeerJ. 2024 Feb 28;12:e17036. doi: 10.7717/peerj.17036 (PMC10908268; doi:10.7717/peerj.17036)
Supplement: Supplemental Information 1 [file peerj-12-17036-s001.pdf]

triact package for R: Analyzing the lying behavior of cows from accelerometer data

Michael Simmler, * Stijn P. Brouwers †

*Digital Production, Agroscope, 8356 Ettenhausen, Switzerland

†Centre for Proper Housing of Ruminants and Pigs, Federal Food Safety and Veterinary Office (FSVO), Agroscope, Tänikon 1, 8356 Ettenhausen, Switzerland

Corresponding author: Michael Simmler; michael.simmler@agroscope.admin.ch; +41 58 469 50 50

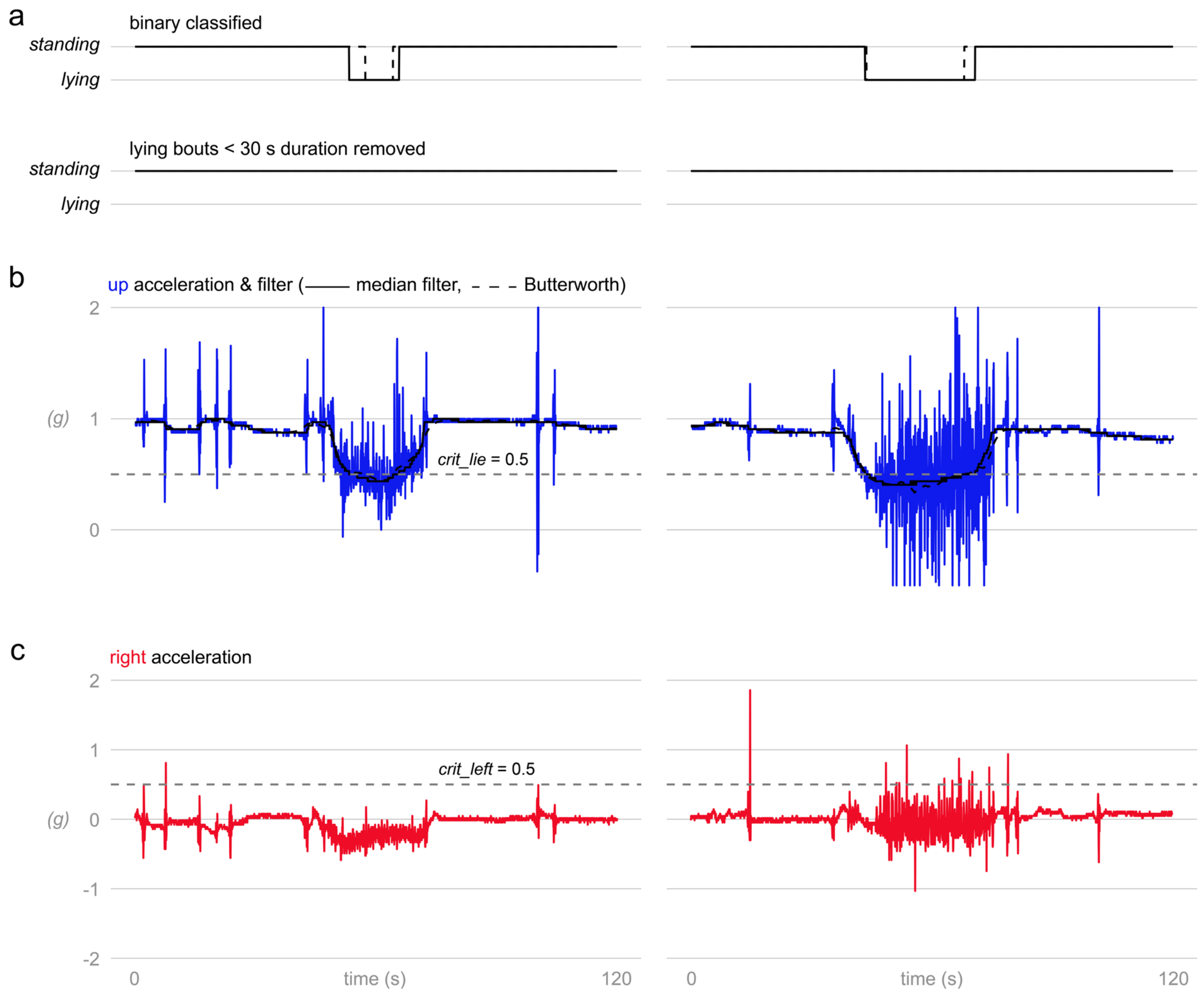

Figure S1. Two examples of a cow lifting the hind leg with the attached accelerometer towards the horizontal position (corresponding to Supplementary Videos 1 and 2). (**a**) Results on standing and lying posture and lying laterality (left L, right R) as determined with *triact*. (**b**) Acceleration as measured on the *upward* axis (up) with filtered data and critical lying threshold indicated as dashed horizontal line. (**c**) Acceleration as measured on the *right* axis with threshold for determining lying side indicated as dashed horizontal line. Data obtained at 20 Hz sampling frequency with a triaxial accelerometer mounted on the left hind leg of a dairy cow.

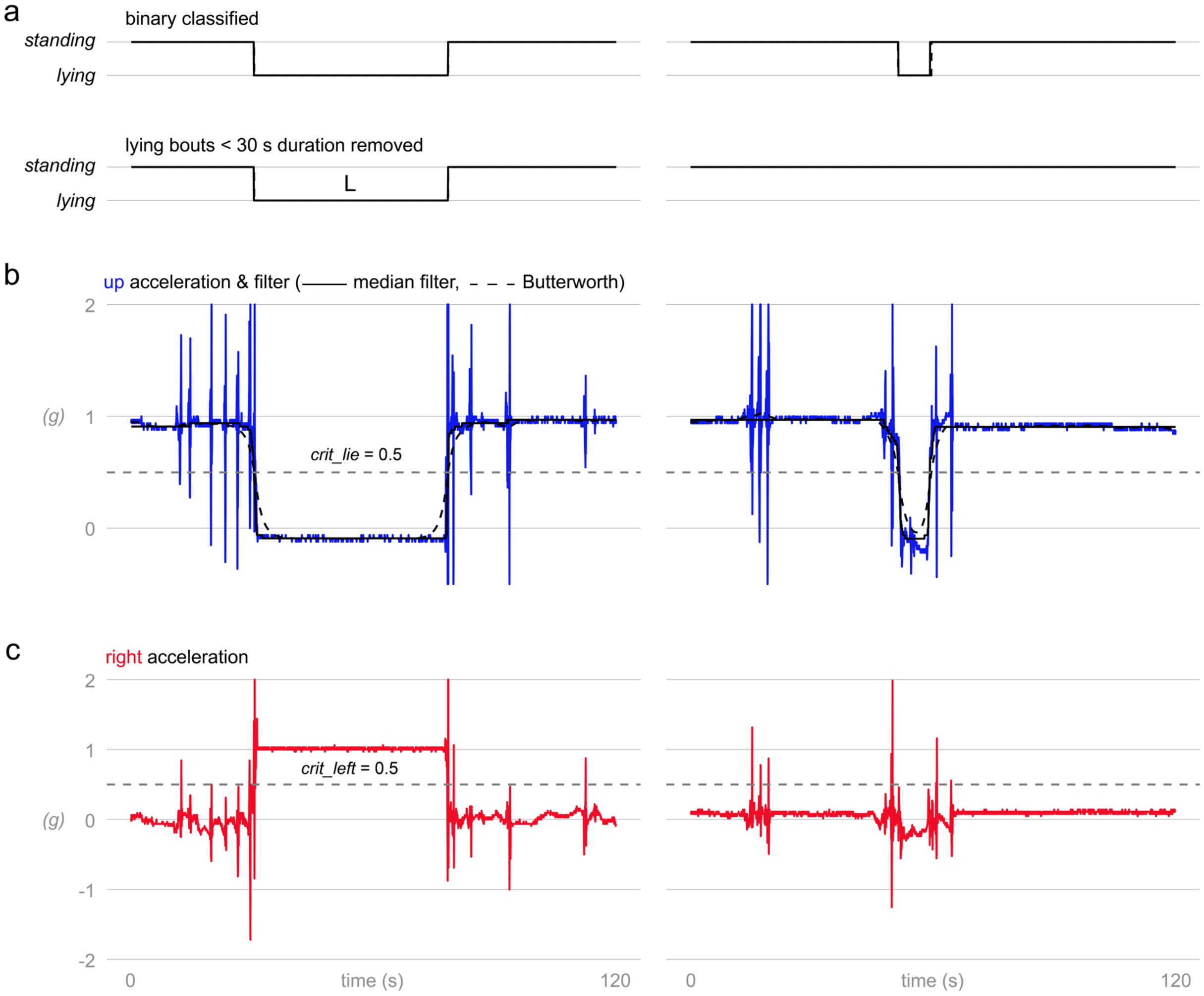

Figure S2. Two examples of short lying bouts (corresponding to Supplementary Videos 3 and 4). The example on the right shows a false negative, as *triact* falsely removed the lying bout in the last step of the algorithm. (**a**) Results on standing and lying posture and lying laterality (left L, right R) as determined with *triact*. (**b**) Acceleration as measured on the *upward* axis (up) with filtered data and critical lying threshold indicated as dashed horizontal line. (**c**) Acceleration as measured on the *right* axis with threshold for determining lying side indicated as dashed horizontal line. Data obtained at 20 Hz sampling frequency with a triaxial accelerometer mounted on the left hind leg of a dairy cow.

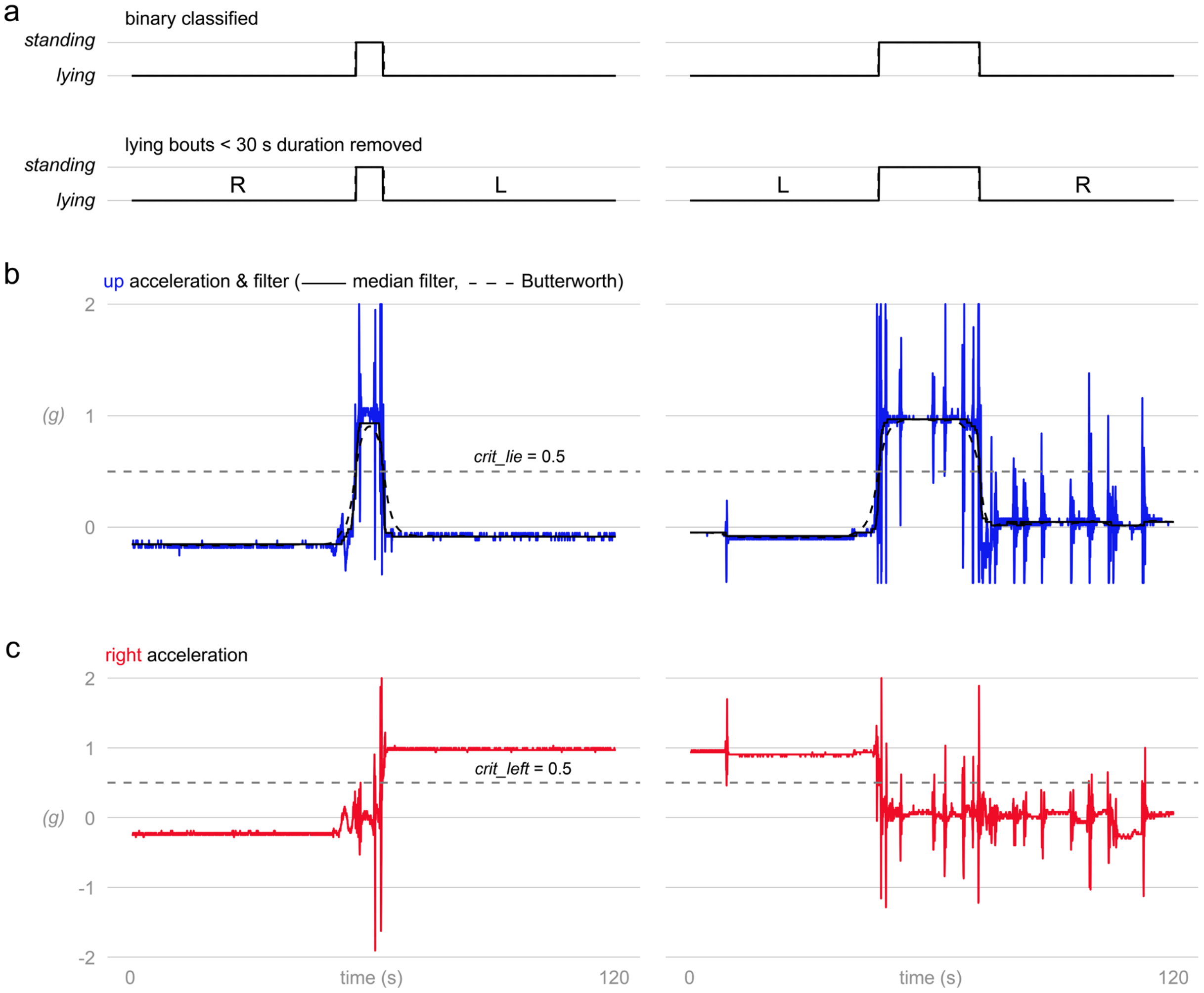

Figure S3. Two examples of short standing bouts (corresponding to Supplementary Videos 5 and 6). (**a**) Results on standing and lying posture and lying laterality (left L, right R) as determined with *triact*. (**b**) Acceleration as measured on the *upward* axis (up) with filtered data and critical lying threshold indicated as dashed horizontal line. (**c**) Acceleration as measured on the *right* axis with threshold for determining lying side indicated as dashed horizontal line. Data obtained at 20 Hz sampling frequency with a triaxial accelerometer mounted on the left hind leg of a dairy cow.
